# The effect of automatic imitation in serial movements with different effectors

**DOI:** 10.3389/fpsyg.2023.1224673

**Published:** 2023-10-18

**Authors:** Hiroshi Ito

**Affiliations:** Department of Psychology, Faculty of Letters, Aichi University, Toyohashi, Japan

**Keywords:** automatic imitation, spatial compatibility, imitative compatibility, serial movement, mirror neuron system

## Abstract

Individuals often imitate the postures or gestures of others in everyday life, without even being aware. This behavioral tendency is known as “automatic imitation” in laboratory settings and is thought to play a crucial role in social interactions. Previous studies have shown that the perception of a simple finger movement activates a shared representation of the observed and executed movements, which then elicits automatic imitation. However, relatively few studies have examined whether automatic imitation is limited to simple single-finger movements or whether it can be produced using a different automatic imitation paradigm with more complex sequential movements. Therefore, this study conducted three experiments in which participants observed the sequential movements of a model and then executed a compatible (similar) action or an incompatible (different) action involving the hand or foot in response to number cues that indicated the sequence for moving their hands or feet. The delay to onset of participants’ initial hand or foot movements was calculated. Participants consistently executed compatible actions faster than incompatible actions. In particular, the results showed an imitative compatibility effect with a human stimulus but not an inanimate stimulus. These results demonstrate that automatic imitation occurs during more complex movements that require memory.

## 1. Introduction

Individuals tend to imitate other people’s postures or gestures in everyday life, often without realizing, and numerous studies in cognitive psychology have suggested that the tendency to imitate is difficult to control (Brass et al., 2000; see Heyes, 2011 and Cracco et al., 2018 for comprehensive reviews). One of the most commonly used tasks to reliably investigate the automatic tendency to imitate (Genschow et al., 2017; Westfal et al., 2021) is the imitation–inhibition task proposed by Brass et al. (2000). In this task, people tend to perform finger lifting faster and more accurately when they observe the same action (compatible trial) rather than a different action (incompatible trial; i.e., the compatibility effect). This automatic tendency to imitate has been reported to occur with body parts other than fingers, such as hands and mouth, and with movements other than lifting, such as opening and closing movements (e.g., Stürmer et al., 2000; Leighton and Heyes, 2010). Furthermore, it also occurs in goal-directed actions involving grasping object with a hand (Catmur and Heyes, 2019).

There is another paradigm called the kinematic paradigm that measures the automatic tendency to imitate (Cracco et al., 2018). In a pioneering study, Kilner et al. (2003) asked participants to move their arm back and forth in the horizontal or vertical plane while observing an experimenter who moved his arm in the parallel (compatible trial) or orthogonal (incompatible trial) plane. In line with the imitation–inhibition task, participants’ movement trajectory contained more variability when the experimenter acted an incompatible movement than when he acted a compatible movement (e.g., Kilner et al., 2007; Roberts et al., 2016).

This kind of imitation is automatic and driven by the fact that the surface features of actions irrelevant to the task at hand encourage similar responses and thwart dissimilar responses (Heyes, 2011). Automatic imitation is distinguished from motor mimicry, which is commonly observed in naturalistic social situations such as conversations because automatic imitation is often observed in well-controlled laboratory situations (Chartrand and Bargh, 1999; Heyes, 2011; Cracco et al., 2018). Although it is unclear whether mimicry and automatic imitation have similar underlying mechanisms (Genschow et al., 2017) and what is measured by reaction time (RT) indices of automatic imitation (Ramsey, 2018), many studies have demonstrated the robustness and reliability of the compatibility effect.

Cognitive neuroscience studies have developed multiple theories of how neural substrates are involved in automatic imitation: the mirror neuron system (see Cook et al. (2014) for a review), the theory of mind network (Frith and Frith, 1999; Saxe and Kanwisher, 2003; Van Overwalle, 2009), and the multiple demand network (see Ramsey, 2018 for a review). Recent work drawing on the imitation–inhibition task showed that activity in the mirror neuron system is modulated by mechanisms that encourage action when humans try to lift their index fingers when another person does the same, but inhibit action when another person lifts a different finger (Cross et al., 2013; Cross and Iacoboni, 2014). However, cognitive neuroscience research has not yet determined how the brain regions indicated by these three theories function together.

Although early studies of imitation assumed that there is an innate imitation mechanism (Meltzoff and Moore, 1977; Meltzoff, 1999), more recent work assumed that automatic imitation arises as a result of general learning and motor control mechanisms (Brass and Heyes, 2005; Cracco et al., 2018). There are two theoretical accounts of these mechanisms: the ideomotor theory (Greenwald, 1970; Prinz, 2005; Massen and Prinz, 2009) and the associative sequence learning (ASL) model (Heyes, 2001, 2005, 2011). Ideomotor theory argues that actions are represented in “common codes,” formed through long-term associative learning between visual and motor representations of action. When we observe another person’s action, it activates a corresponding motor representation in the observer (Brass and Heyes, 2005), leading to automatic imitation. In contrast to ideomotor theory, the ASL model does not assume anticipatory representations of the sensory consequences of an action (Heyes, 2011). Instead, this model argues that experiences of observing and executing an action lead to bidirectional connections between the perceptual and motor representations of the action through long-term associative learning, which then leads to automatic imitation. Despite the differences, these two theories are similar in that they attempt to explain automatic imitation through a common representation of observed and executed actions.

Although the robustness of automatic imitation has been well-established using the imitation–inhibition task in particular over the past 20 years, important theoretical questions remain (Cracco et al., 2018). Several questions have been raised regarding the mechanisms underlying automatic imitation. For instance, what is the origin of automatic imitation in imitation–inhibition tasks? Other questions are linked to factors that modulate automatic imitation. For instance, what is the role of memory in a sequence of movements in automatic imitation in an imitation–inhibition task?

**Figure 1:**
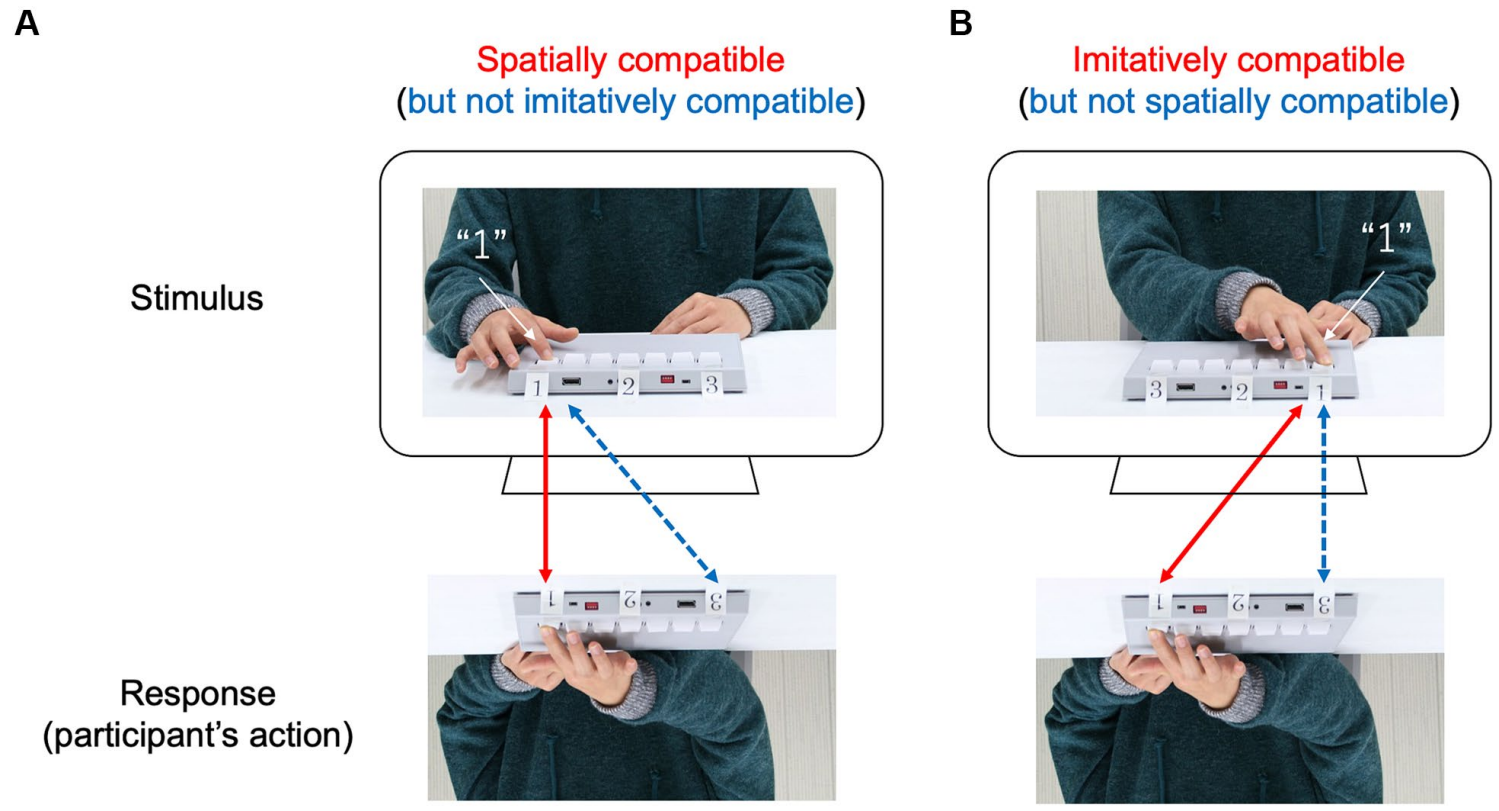
Diagram of the two compatibilities. A model (i.e., stimulus) presses a sequence of keys (e.g., 1-2-3) on the keypad with her right index finger, and the participant is instructed to imitate the model. **(A)** Spatially compatible trial: the keys are oriented so that pressing the sequence requires the participant and model to press in the same locations in space, but to do so means the participant’s right index finger and the model’s right index finger move anatomically different. **(B)** Imitatively compatible trial: the pressing task demands that the participant’s right index finger and the model’s right index finger move in a different direction, however, their movements are anatomically the same.

To determine the origin of automatic imitation in the imitation–inhibition task, we must distinguish between two ways in which the actions of participants could be compatible with the actions of a model actor (i.e., spatial compatibility and imitative compatibility; Figure 1). Spatially compatible trials emphasize that the actions of participants are directed to the same location in space but not to the absolute imitation of the model. For example, participants can move their arms faster (a response on the left side of space) when the cue to move is accompanied by an image of an action performed on the left side of space compared to the right side of space (Figure 1). In contrast, imitatively compatible trials emphasize that the actions of participants are performed as anatomically same arm movements (i.e., absolute imitation), but without spatial compatibility. For example, participants can move their arms faster when the cue to move is accompanied by an image of an anatomically identical action using the same arm with the same trajectory profile, even if the actions of participants are directed to a different location in space. If automatic imitation is independent of spatial compatibility and is produced by the anatomical similarity of movements between the model and the participant, one would expect responses to be faster in imitatively compatible trials (but not in spatially compatible trials) than in imitatively incompatible trials.

Three methods have been developed to examine the extent that automatic imitation depends on spatial compatibility (Cracco et al., 2018). The first method is to position the stimulus hand orthogonal (i.e., rotating the stimulus hand 90° counterclockwise) to the response hand (e.g., Heyes et al., 2005; Santiesteban et al., 2012). However, this method has a potential issue that there is a documented tendency to associate “up” with “right” and “down” with “left” (Weeks and Proctor, 1990). Consequently, this method confounds automatic imitation with orthogonal spatial compatibility (Heyes, 2011). To address this problem, the second method have been proposed by separately manipulating spatial and imitative compatibility (e.g., Catmur and Heyes, 2011; Boyer et al., 2012; Sowden and Catmur, 2015). For example, considering the hand stimulus used by Brass et al. (2000), this separation is achieved by presenting a left stimulus hand in one half of the trials and a right stimulus hand in the other half of the trials. This experimental setup results in a positive relationship between spatial and imitative compatibility in left hand trials and a negative relationship in right hand trials, which makes it possible to calculate a main effect of imitative compatibility that is independent of spatial compatibility (Cracco et al., 2018). Finally, the third method addresses the spatial compatibility confound by using more complex stimuli such as symbolic gestures that cannot easily be categorized on a simple spatial dimension (e.g., Liepelt et al., 2010; Bortoletto et al., 2013). Although many studies have demonstrated that imitative compatibility is present regardless of spatial compatibility, an open question still remains how much of automatic imitation can be explained by spatial process because it is difficult to completely eliminate spatial compatibility (Cracco et al., 2018). Therefore, in this study, automatic imitation was examined with attention paid to these two compatibilities.

In our everyday motor learning, it is well-know that people pay attention to observed actions, remember them, and then reproduce those actions (Bandura, 1986; Ste-Marie et al., 2012). Through this process, people are learning more complex sequences of movements from others. One study using sports movements, such as darts, have reported that when experts observed actions of novices and repeatedly predicted the darts scores of them, the darts performance of the experts deteriorated after this observation (Ikegami and Ganesh, 2014). This result suggests that automatic imitation occurs in a social context. However, a few studies have examined whether automatic imitation occurs when participants performed movements after observing others in a delay paradigm. Therefore, regarding the second question, the role of memory in the sequence of movements during automatic imitation is worth considering.

The role of memory in automatic imitation can be inferred from the results of studies on implicit memory (e.g., Graf and Schacter, 1985; Schacter, 1987; Roediger, 2003; Mitchell et al., 2018) or priming effect (e.g., Neely, 1977; Parkin and Russo, 1990). Implicit memory refers to the phenomenon in which prior experiences influence performance in tasks that do not require conscious or intentional recollection of those prior experiences. Considering that the imitative compatibility effect would arise from a process in which observing an action automatically activates a motor representation similar to the observed action (Heyes, 2011), there is a potential for the activated representation to be retained as a form of implicit memory. This suggests that visual representations associated with observed bodily movements of others might modulate subsequent self-generated motor actions. Therefore, if, when introducing the delay paradigm, self motor representations and other motor representations automatically competes in a manner similar to the traditional automatic imitation paradigm, it is anticipated that the imitative compatibility effect will occur in this type of paradigm, as well.

Although automatic imitation is well-established in the imitation–inhibition task, most studies have demonstrated the effects using minimal and simple stimuli, such as simple finger or hand movements performed by isolated hands, in limited contexts (Heyes, 2005; Bertenthal et al., 2006; Catmur and Heyes, 2011, 2019; Cook and Bird, 2011; Boyer et al., 2012). If the degree that automatic imitation occurs when methods incorporate more complex movements with a whole-body can be examined, these methods can provide a bridge between the minimal automatic imitation tasks and real-life social psychology mimicry tasks. Here, “complex movements” includes sequences of movements that require people to remember kinematic characteristics—for example, changing their hand-and-arm positions, or different movement pathways.

In one study, participants were asked to perform sequential hand-and-arm movements that were compatible or incompatible with the actions they observed a life-sized 3D virtual character performing (Pan and Hamilton, 2015). The participants were initially asked to observe a virtual character performing sequences of tapping on three drums on a table from a third-person perspective. They were then asked to execute compatible or incompatible movements in response to number cues on the screen that indicated the order of tapping on the three drums. Compatibility was manipulated in two ways: participants were instructed to move their arms to the same relative locations as the stimulus (a spatially compatible response) or to execute anatomically the same arm movements (an imitatively compatible response). Participants responded more quickly when actions were spatially compatible rather than imitatively compatible, and when actions were imitatively compatible rather than imitatively incompatible. These results suggest that the imitative compatibility effect can be seen in sequential hand-and-arm movements that require memorization of kinematic characteristics. Therefore, automatic imitation can occur when people perform the same or different movements while watching another person’s movements and after watching them. If the observation of another person’s movements activates the same response representation in an observer, the memory of the other person’s movements could work to maintain activation and support the same response, even if there is a time lag. Such a process saves time; therefore, responses are faster when people attempt to perform the same actions after watching another person’s actions. Moreover, automatic activation of memory of another person’s actions has the potential to play a crucial role when people try to imitate and learn such actions in everyday life. However, if the observation of another person’s movements automatically activates a response representation that is different from that intended by the observer, this automatic activation of the movements must be inhibited for the observer to perform the correct response.

One study that used a hand-and-arm movement task (Pan and Hamilton, 2015) suggested that people automatically imitate compatible movements performed by virtual others. Using virtual characters to examine the imitative compatibility effect in a well-controlled laboratory setting could be worthwhile. However, it is unclear whether automatic imitation is limited to simple single finger or hand movements, or whether it can be produced in a different automatic imitation paradigm in which more complex movements performed by a *real human* are to be imitated.

There is extensive literature on whether automatic imitation is sensitive to the similarity between the actor and imitator; that is, the imitator resembles the actor in appearance (e.g., Chaminade and Cheng, 2009; Press, 2011; Cracco et al., 2018). For example, Cracco et al. (2018) indicated the presence of a linear trend with stronger automatic imitation for human versus non-human model such as robots (e.g., Press et al., 2006), and stronger automatic imitation for non-human model than for geometric models such as moving dots (e.g., Gowen et al., 2016) in their meta-analysis. However, it is still controversial that there is a clear-cut human bias in automatic imitation (e.g., Klapper et al., 2014). Thus, this study determined whether automatic imitation occurs in more natural settings with human agents and more realistic motion profiles.

In this study, three experiments were conducted, each with an observation-execution task similar to that of Pan and Hamilton (2015). Participants saw either serial hand-and-arm movements (Experiment 1) or foot-and-leg movements (Experiment 2) performed by a human, and then executed compatible or incompatible movements. This study varied whether the observed movements were spatially or imitatively compatible, and whether the participant’s response was compatible or incompatible. It was expected that people would respond more quickly in compatible trials than in incompatible trials for both spatial and imitative movements. Finally, to address whether human forms and biological movements are crucial for this effect, Experiment 3 was conducted. In Experiment 3, participants saw three lights that indicated the same goal locations and sequences of responses as the human model, but without any human form or biological movement. If there is a strong social basis for imitation, one would expect the imitative compatibility effect to be weak or absent under these conditions.

## 2. Experiment 1

### 2.1. Method

#### 2.1.1. Participants

The required sample sizes for all three experiments were determined using G*Power software (Faul et al., 2007). All power analyses were performed assuming desired power of 80% at alpha = 0.05 and were based on typical small, medium, and large effect sizes (Cohen’s f): 0.10, 0.25, and 0.40, respectively (Cohen, 1992). These effect sizes corresponded to partial η^2^ values of approximately 0.01, 0.06, and 0.14, respectively. For repeated-measures analyses of variance (ANOVAs), the required sample sizes for small, medium, and large effect sizes were 138, 24, and 12, respectively. Based on these considerations, a sample size of 24 participants was deemed necessary to detect medium-sized effects. The sample size was increased to 29 to account for the risk of participants dropping out of the study or obtaining invalid or missing data (e.g., due to technical problems).

Participants in Experiment 1 were 29 undergraduate students (22 females and 7 males, M_age_ = 21.4 years, SD = 1.8 years, age range: 20 to 30 years) taking a psychology course at A University in Japan. All participants were right-handed based on the Edinburgh Handedness Inventory (Oldfield, 1971), which has been translated into Japanese by native speakers of Japanese with good knowledge of English. The participants had normal or corrected-to-normal vision, were naïve to the study purpose, and received prepaid cards for purchasing books (1,000 JPY) for taking part. This experiment, and all other experiments were conducted according to the principles and guidelines of the Declaration of Helsinki and were approved by the ethics board of Aichi University. All participants provided written informed consent.

#### 2.1.2. Design

A 2 (type of compatibility: spatial or imitative) × 2 (trial type: compatible or incompatible) within-subjects design was used.

#### 2.1.3. Procedure

**Figure 2:**
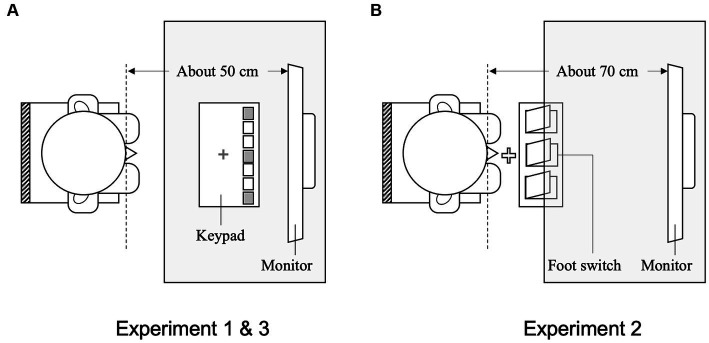
Schematic drawings of the overhead view of setups in Experiments 1, 2, and 3. **(A)** Hand response setup in Experiment 1 and 3: participants were seated on a chair with their right index fingers placed on a red cross marked on the response keypad. The position of the chair was adjusted to a natural height for each participant. The viewing distance from the monitor was approximately 50 cm. **(B)** Foot response setup in Experiment 2: participants were seated on a chair without shoes and placed their right great toe on a white cross marked on the floor. The position of the chair was adjusted to a natural height for each participant. The viewing distance from the monitor was approximately 70 cm.

All participants were tested individually in a laboratory. Each participant observed the sequential movements of a model and then executed a compatible (similar) action or an incompatible (different) action involving the hand in response to number cues. The participants were seated on a chair, and their right index fingers were placed on a red cross marked at the center of the response keypad (RB-740, Cedrus Co., United States), which was fixed on the table. They used only three of the seven keys (rightmost, middle, and leftmost) on the response keypad for their responses. The participants were instructed to keep their eyes on the monitor and observe the target movies. Figure 2A illustrates the equipment setup. Stimuli were displayed on a 19-inch-wide LCD monitor (EPSON, Japan) located at a viewing distance of approximately 50 cm.

Before participating in the experimental trials, participants completed four practice trials. Each experimental session consisted of two blocks of trials. Each type of compatibility (i.e., imitative and spatial conditions) was conducted in a separate block. The order of the two experimental blocks was counterbalanced across participants, resulting in approximately equal numbers of participants in each group (imitative-first group: 15; spatial-first group: 14). Each experimental block consisted of 48 trials, which were presented in random order. In each of the imitative and spatial conditions, half of the trials (24 trials) were assigned to the compatible set, in which an action compatible with the actor was required, and the other half (24 trials) were assigned to the incompatible set, in which an action incompatible with the actor was required.

The sequences consisted of the numbers 1, 2, and 3, excluding sequences in which the same numbers were presented two or three times (e.g., “1, 1, 1” and “1, 2, 2”). Six possible combinations (i.e., “1, 2, 3,” “1, 3, 2,” “2, 1, 3,” “2, 3, 1,” “3, 2, 1,” and “3, 1, 2”) were used—each of which was repeated four times in the block. In the compatible trials, the actor pressed the same sequence of keys as the participant. In contrast, in the incompatible trials, the actor pressed a different sequence from that of the participant. As in the compatible trials, each of the six target sequences was repeated four times.

**Figure 3:**
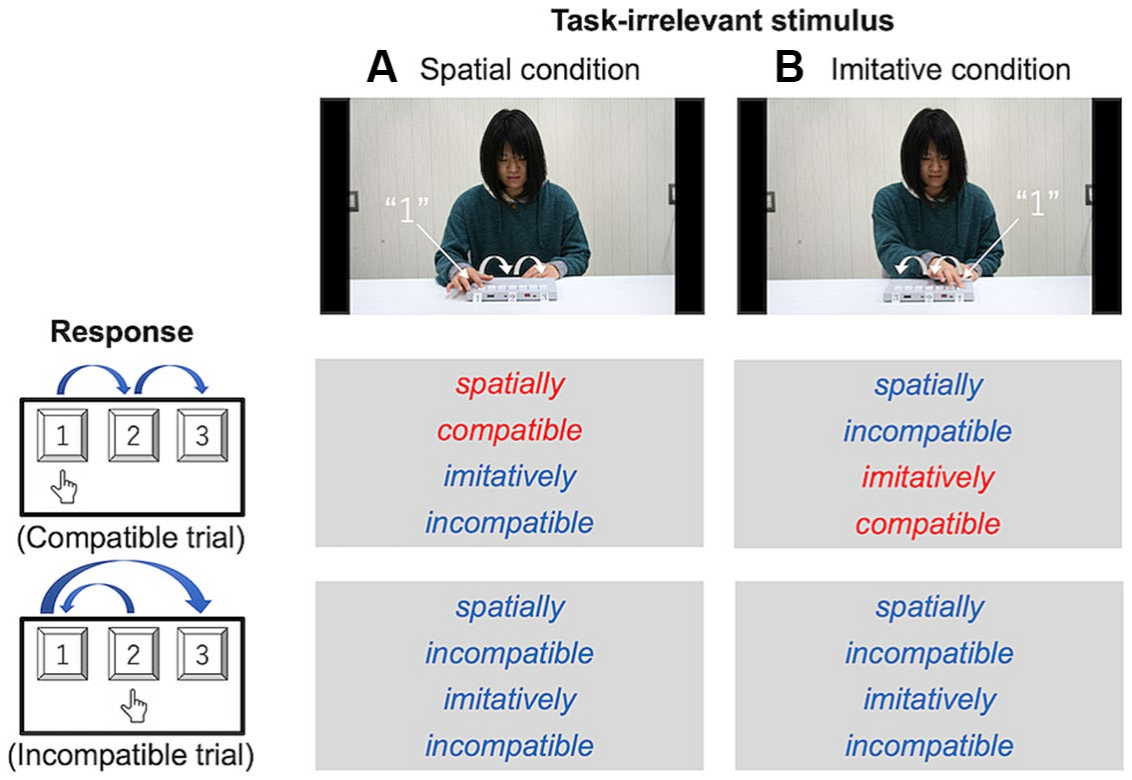
Schematic configurations of the spatial **(A)** and imitative **(B)** conditions in Experiment 1. In the spatial condition, participants’ responses and the keypad numbering were spatially compatible between the actor in the movies and the participant (e.g., in the compatible trials of the spatial condition, participants were asked to press keys at the same locations in space as did the actor in the movies). In the imitative condition, these were imitatively compatible between the actor and participant (e.g., in the compatible trials of the imitative condition, participants were asked to move their right arm in a similar kinematic way; that is, using a similar trajectory profile, as the action of the actor’s right arm, regardless of the key’s location in space).

Figure 3 illustrates the movement scenes for each type of compatibility. In the spatial condition, the actor pressed the keys at the same locations in space and in the same order as the participants in the compatible trials. In contrast, the actor pressed the keys at different locations in space and with different movements (i.e., different trajectories) in the incompatible trials. The incompatible trials were designed such that the movement of the actor was spatially and imitatively incompatible. In the imitative condition, the actor pressed keys with the same movements (i.e., the same trajectories) in the same order as the participants in the compatible trials. In contrast, the actor pressed the keys at different locations in space and with different movements in incompatible trials, as well as in the spatial condition.

**Figure 4:**
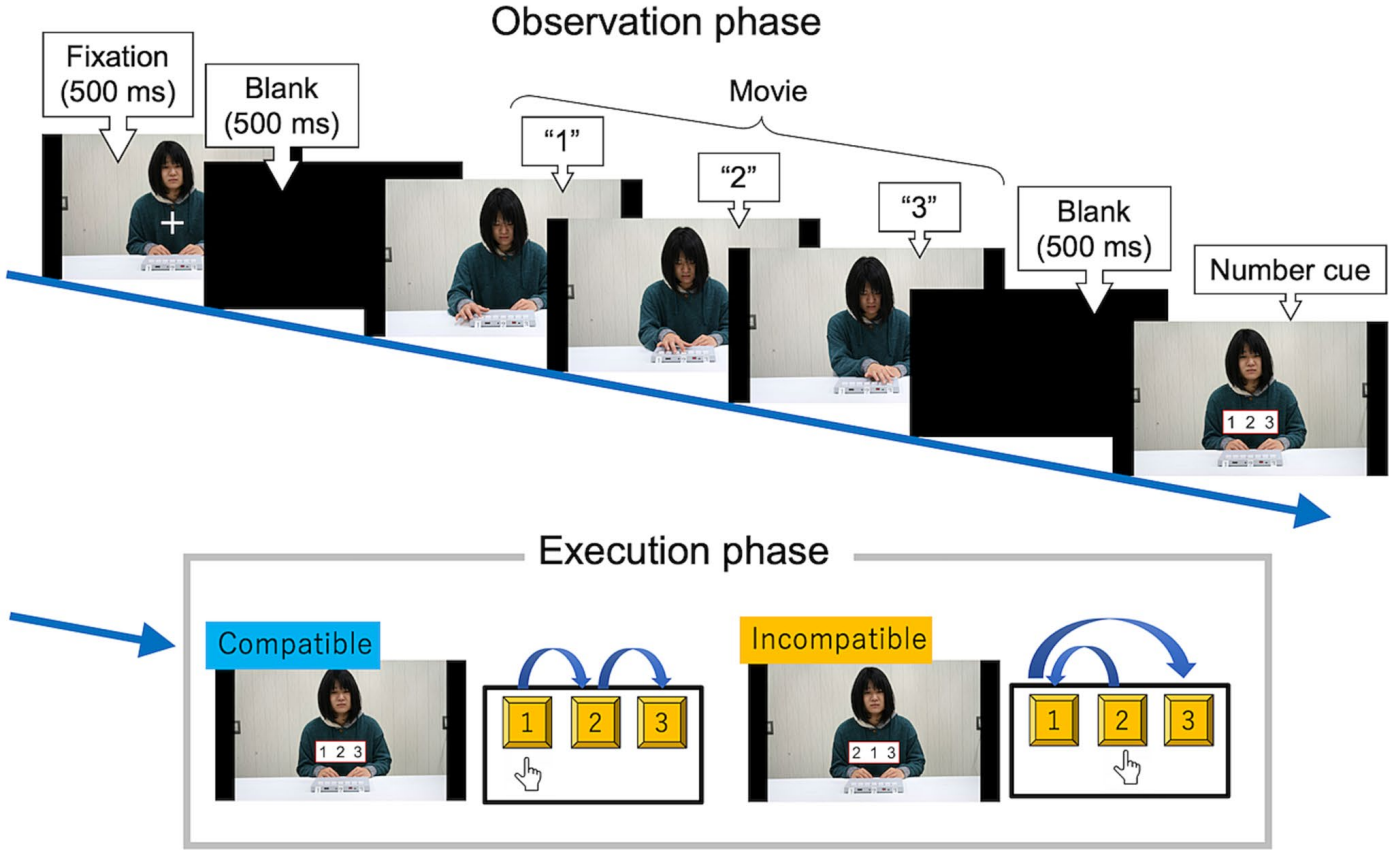
Stimulus presentation schedule of the observation-execution task in Experiment 1. Participants were asked to observe a serial hand-and-arm movement and then press each key in the same sequence as the number cue on the monitor as quickly and accurately as possible. In this example, the participant’s correct response (“1, 2, 3”) was spatially compatible between actor and participant.

Figure 4 shows the stimulus presentation schedule for each trial in Experiment 1. The trial consisted of two phases: an observation phase and an execution phase.

In the observation phase, the target movies appeared. Each movie was of resolution 788 × 1,400 pixels, and lasted five seconds without audio. The three keys were labeled as 1, 2, and 3. In the spatial condition, the keys in the movies were numbered 1, 2, and 3 from left to right on the screen. In the imitative condition, the keys in the movies were numbered in the opposite order (i.e., 3, 2, and 1 from left to right of the screen). After a blank interval, a number cue appeared. The number cue remained until the participants pressed any of the three keys.

In the execution phase, participants were asked to press the three number keys with their index finger in the order of the number cue on the monitor as quickly and accurately as possible and then return to the resting position. They were instructed that some of the observed sequences matched the cued sequences, whereas others did not. They received no feedback on the accuracy of their responses. After the response, there was an intertrial interval of five seconds.

After the first experimental block, there was a one-minute rest period. Each experimental block lasted for approximately 12 min, depending on the participants’ pace, and the experimental session was completed in less than 30 min.

A motion-capture system (OptiTrack Japan, Ltd.) was employed to estimate the motion profiles of the participants’ responses. This system consisted of six cameras (Prime 13, OptiTrack Japan, Ltd.) that recorded the raw position data of the active infrared markers from the fingertips of the participants’ right index fingers. The sampling rate of the cameras was 240 frames per second (fps) with images of 1.3 megapixels resolution. Thus, motion profile data in a three-dimensional space was obtained. The motion-capture system was connected to a synchronization unit (eSync 2, OptiTrack Japan, Ltd.), and Transistor-Transistor Logic (TTL) signals were output from this unit as external triggers. The TTL signals were then input into a motion-capture system to record the timing of the number cue presentation on the motion-capture data.

After the experiment, the RT was calculated; that is, the time from the onset of the number cue to the onset of the initial hand movements of the participants. The movement onsets were calculated offline using MATLAB R2017b (MathWorks, United States). The movement onset was defined as the point at which the velocity of the finger marker exceeded the relative velocity threshold, which was 10% of peak velocity (Brass et al., 2000).

### 2.2. Results and discussion

Experiment 1 examined the extent that automatic imitation of human stimuli depends on spatial compatibility and the role of memory in automatic imitation of sequential hand-and-arm movements. RTs for error trials in which participants pressed the keys in a different order than that indicated by the number cues (0.6% of data), or in which the RTs were ± 3 from each participant’s mean (1% of data), were excluded from further analysis. Data from 29 participants were analyzed.

**Figure 5:**
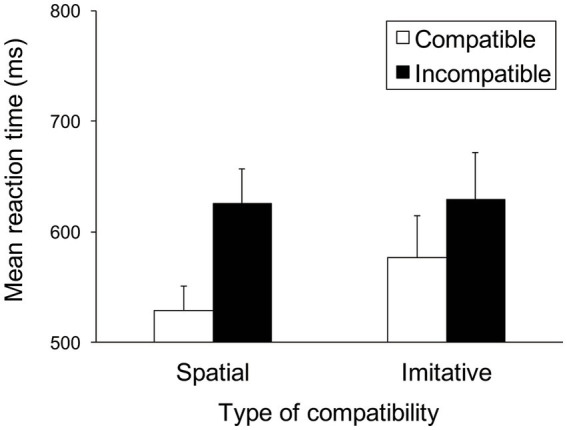
Mean reaction times (ms) in Experiment 1 for each type of compatibility and for each trial type. Error bars represent standard errors.

Figure 5 shows the mean RTs for each trial type in the spatial and imitative conditions. A 2 (spatial and imitative) × 2 (compatible and incompatible) repeated-measures ANOVA revealed a significant main effect of trial type (compatible vs. incompatible), *F*(1, 28) = 24.39, *p* < 0.001, *η_p_*^2^ = 0.466, and a significant interaction between type of compatibility and trial type, *F*(1, 28) = 8.43, *p* < 0.01, *η_p_*^2^ = 0.232, but no significant main effect of type of compatibility (spatial vs. imitative), *F*(1, 28) = 1.38, *p* = 0.251, *η_p_*^2^ = 0.047.

A simple main-effects analysis of the interaction showed that the mean RT in the compatible trial was significantly faster than that in the incompatible trial (compatible vs. incompatible), both in the spatial and imitative conditions, *F*(1, 28) = 31.47, *p* < 0.001, *η_p_*^2^ = 0.529 and *F*(1, 28) = 10.13, *p* < 0.01, *η_p_*^2^ = 0.266, respectively. Additionally, there was a significant simple main effect of type of compatibility (spatial vs. imitative) in the compatible trial, *F*(1, 28) = 4.31, *p* < 0.05, *η_p_*^2^ = 0.134. This simple main effect showed that the mean RT in the spatial condition was significantly faster than that in the imitative condition in the compatible trial. However, no significant simple main effect of type of compatibility (spatial vs. imitative) was found in the incompatible trial, *F*(1, 28) = 0.03, *p* = 0.870, *η_p_*^2^ = 0.001.

The mean error proportion for each type of compatibility and each trial type was extremely low (< 1%). The error data were subjected to a repeated-measures ANOVA with the same factors as the RT data. The results showed no significant main effects or interactions, *F*(1, 28) < 1, *p* > 0.48, *η_p_*^2^ < 0.013.

Faster RTs were found with compatible actions in the imitative condition, a finding consistent with Pan and Hamilton’s (2015) results for serial movement. These results extend Pan and Hamilton’s (2015) study by suggesting that imitative compatibility can be obtained when observing a more natural human stimulus, particularly in the case of sequential hand-and-arm actions that require the memory of kinematic characteristics, such as a change in position and movement pathways. The imitative compatibility effect may indicate that a participant’s hand response was unintentionally influenced by the observed hand-and-arm movements.

Furthermore, the results showed that RTs in the spatial condition were faster than those in the imitative condition but only when the participant’s hand response was compatible with the observed movement. These results suggest that the effect of compatibility was greater when the hand response was spatially consistent with the observed movement rather than imitatively consistent with it. Such a pattern may indicate independence between spatial and imitative compatibility, suggesting that automatic imitation is not merely a byproduct of spatial processing. This notion is consistent with previous studies that reported independence of imitative compatibility (Heyes, 2005; Bertenthal et al., 2006; Catmur and Heyes, 2011, 2019; Cook and Bird, 2011; Boyer et al., 2012).

## 3. Experiment 2

Experiment 1 showed that imitatively compatible responses were faster than incompatible ones. This finding raises the question of whether imitative compatibility is specific to hand-and-arm responses. If the imitative compatibility effect is produced by a long-term associative link between sensory and motor representations of the same action (Brass and Heyes, 2005; Heyes, 2011), one would expect that it could be also obtained in any response method related to whole-body movement (i.e., in different parts of the body; “effectors”). Thus, the aim of Experiment 2 was to determine how well the pattern of results in Experiment 1 would be replicated when a foot-and-leg response was required instead of a hand-and-arm response.

### 3.1. Method

#### 3.1.1. Participants

Participants in Experiment 2 were 29 undergraduate students (21 females and 8 males, M_age_ = 20.9 years, SD = 0.4 years, age range: 20 to 22 years) taking a psychology course at A University in Japan. None of the participants participated in Experiment 1. I determined the participants’ dominant foot by asking, “Which foot do you usually use, for example, to kick a ball?” All participants were right-footed, had normal or corrected-to-normal vision, and were naïve to the study purpose. They received prepaid cards to purchase books (1,000 JPY) for taking part.

#### 3.1.2. Design

I used the same experimental design as described in Experiment 1.

#### 3.1.3. Procedure

**Figure 6:**
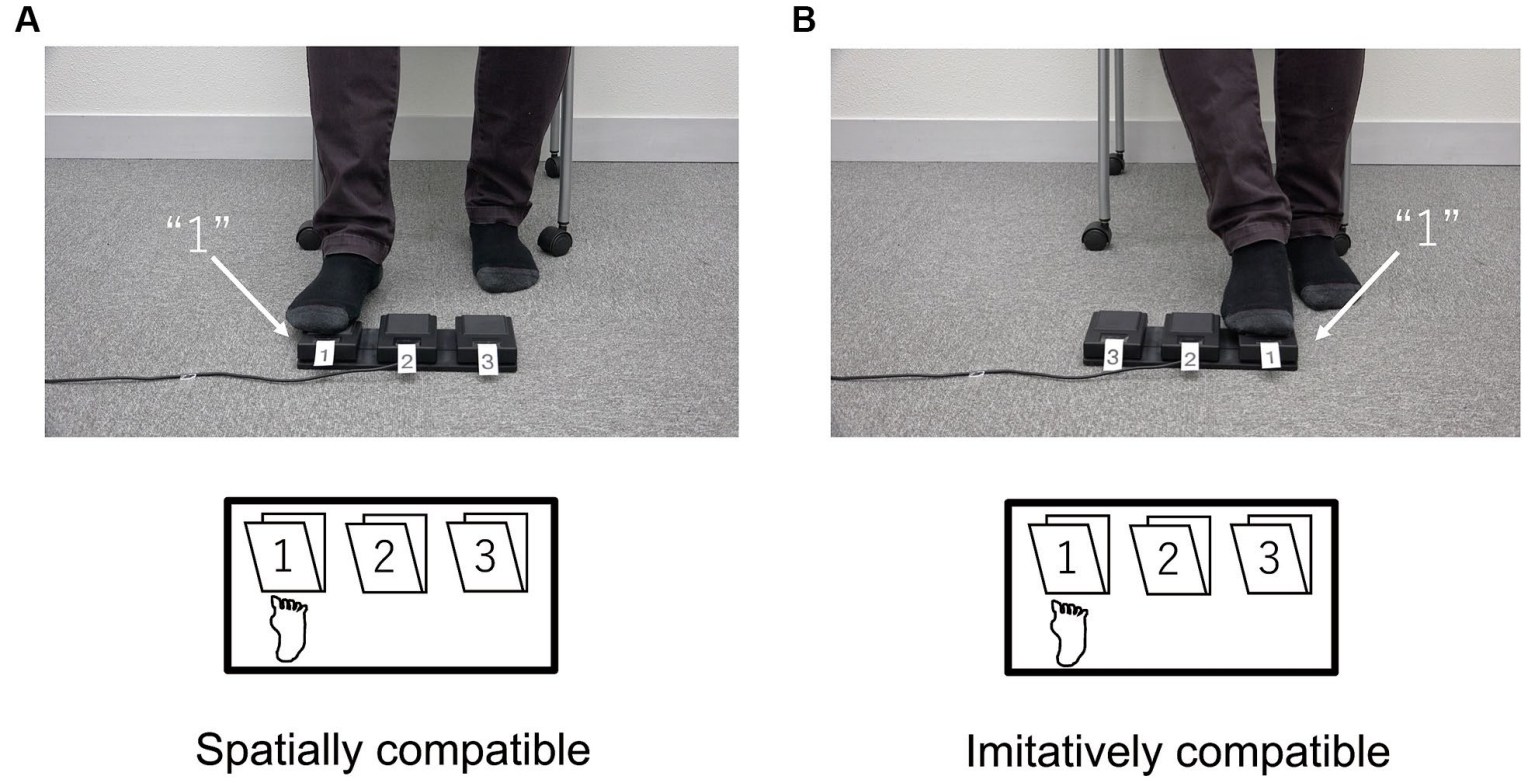
Schematic configurations of the spatial **(A)** and imitative **(B)** conditions in Experiment 2. In the spatial condition, participant’s responses and the triple-foot switch numbering were spatially compatible between the actor in the movies and participant. In the imitative condition, these were imitatively compatible between the actor and participant.

The task and procedure in Experiment 2 were the same as those in Experiment 1, except that they were modified for foot responses. The participants were seated on a chair and their right great toe was placed on a white cross marked on the floor. Figure 2B illustrates the equipment setup used in Experiment 2. A triple-foot switch (FS1P3 Foot Switch Triple, EDIKUN Co., Japan) was fixed to the floor approximately 5 cm in front of the white cross. The three pedals on the triple-foot switch on the floor and the three pedals in the movies were labeled 1, 2, and 3. Figure 6 illustrates each type of compatibility (spatial and imitative).

The stimuli shown to the participants in Experiment 2 consisted of a set of sequential foot-and-leg movements. In each target stimulus, the actor performed a sequence of stepping on three pedals on the floor (e.g., “2, 3, 1”). Each movie was 788 × 1,400 pixels in resolution and lasted five seconds without audio. After the appearance of the target stimulus, participants were asked to step on each foot pedal in the same order as the number cue on the monitor, as quickly and accurately as possible, and then return to the resting position. Experiment 2 had approximately equal numbers of participants in each group (imitative-first group: 15; spatial-first group: 14).

The apparatus and calculation of RTs in Experiment 2 were the same as those in Experiment 1; however, the equipment setup for the foot responses was modified. The raw position data of the active infrared markers from the tip of the participant’s right great toe were recorded.

### 3.2. Results and discussion

Experiment 2 investigated whether an imitative compatibility effect could be observed in different parts of the body. RTs for error trials in which participants stepped on the pedals in a different order than that indicated by the number cues (0.5% of data), or in which the RTs were ± 3 from each participant’s mean (0.5% of data), were excluded from further analysis. Data from 29 participants were analyzed.

**Figure 7:**
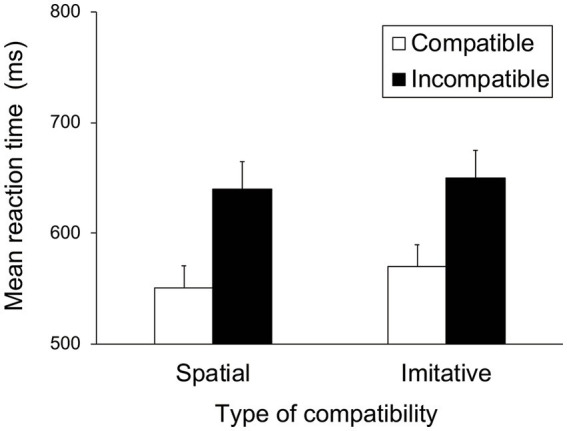
Mean reaction times (ms) in Experiment 2 for each type of compatibility and for each trial type. Error bars represent standard errors.

Figure 7 shows the mean RTs for each trial type in the spatial and imitative conditions. A 2 (spatial and imitative) × 2 (compatible and incompatible) repeated-measures ANOVA revealed a significant main effect of trial type (compatible vs. incompatible), *F*(1, 28) = 35.40, *p* < 0.001, *η_p_*^2^ = 0.558, but no significant main effect of type of compatibility (spatial vs. imitative) or interaction between type of compatibility and trial type, *F*(1, 28) = 1.58, *p* = 0.220, *η_p_*^2^ = 0.053, and *F*(1, 28) = 0.720, *p* = 0.403, *η_p_*^2^ = 0.025, respectively. These results revealed that the mean RT in the compatible trial was faster than that in the incompatible trial in both the spatial and imitative conditions. This suggests that the observation of sequential foot-and-leg movements elicits both spatial and imitative compatibility effects.

The mean error proportion for each type of compatibility and each trial type was extremely low (< 1%). The error data were subjected to a repeated-measures ANOVA with the same factors as the RT data. The results showed no significant main effects or interactions, *F*(1, 28) < 1.29, *p* > 0.26, *η_p_*^2^ < 0.043.

## 4. Experiment 3

Experiment 1 showed that imitatively compatible responses facilitated RTs more than incompatible responses did. This finding raises the question as to whether imitative compatibility is specific to human-like stimuli (i.e., a human form with biological movement). If there is indeed a strong social basis for imitation, one would expect that the imitative compatibility effect would be weak or absent in response to a non-human stimulus, such as inanimate objects or light-indicating sequences. Thus, Experiment 3 aimed to determine whether human stimuli are crucial for the imitative compatibility effect.

### 4.1. Method

#### 4.1.1. Participants

Participants in Experiment 3 were 29 undergraduate students (21 females and 8 males, M_age_ = 21.1 years, SD = 0.7 years, age range: 20 to 23 years) taking a psychology course at A University in Japan. None of the participants had participated in Experiments 1 or 2. All participants were right-handed, based on the Japanese translation of the Edinburgh Handedness Inventory (Oldfield, 1971) used in Experiment 1. The participants had normal or corrected-to-normal vision, were naïve to the study purpose, and received prepaid cards for purchasing books (1,000 JPY) for taking part.

#### 4.1.2. Design

I used the same experimental design described in Experiment 1.

#### 4.1.3. Procedure

**Figure 8:**
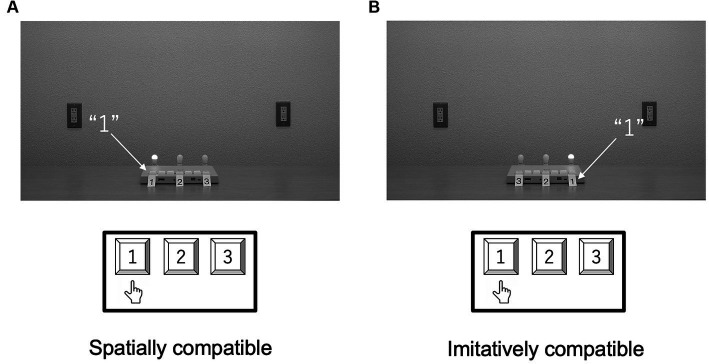
Schematic configurations of the spatial **(A)** and imitative **(B)** conditions in Experiment 3. In the spatial condition, participants’ responses and the keypad numbering were spatially compatible with the light in the movies. In the imitative condition, the light and participant were imitatively compatible.

The task of the participants and the procedure in Experiment 3 were the same as those in Experiment 1, except that the sequences were indicated with lights. Figure 8 shows each type of compatibility condition.

The stimuli shown to participants in Experiment 3 consisted of a set of movies, in each of which a light turned on and off one by one in a sequential manner (e.g., “2, 3, 1”). Each movie was 788 × 1,400 pixels in resolution and lasted for five seconds without audio. After the appearance of the target stimulus, participants were asked to press each key in the same order as the number cue on the monitor as quickly and accurately as possible and then return to the resting position. Experiment 3 had approximately equal numbers of participants in each group (imitative-first group: 15; spatial-first group: 14).

The apparatus and calculation of the RTs in Experiment 3 were the same as in Experiment 1. Raw position data of the active infrared markers were recorded from the participants’ right index fingertips.

### 4.2. Results and discussion

Experiment 3 investigated whether the imitative compatibility effect was observed in response to an inanimate object. RTs for error trials in which participants pressed the keys in a different order than that indicated by the number cues (0.6% of data) or in which the RTs were ± 3 from each participant’s mean (0.4% of data) were excluded from further analysis. Data from 29 participants were analyzed.

**Figure 9:**
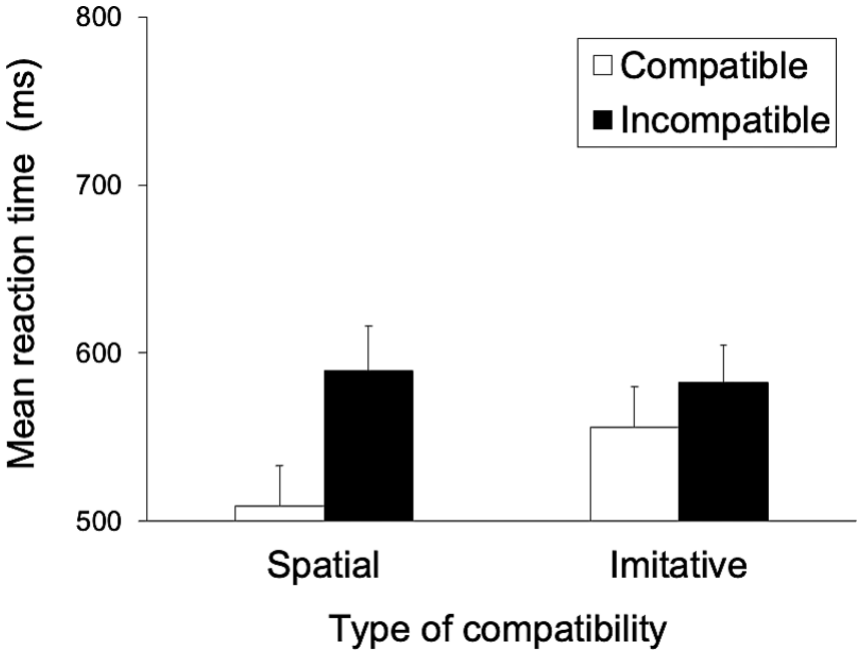
Mean reaction times (ms) in Experiment 3 for each type of compatibility and for each trial type. Error bars represent standard errors.

Figure 9 shows the mean RTs for each trial type in the spatial and imitative conditions. A 2 (spatial and imitative) × 2 (compatible and incompatible) repeated-measures ANOVA revealed a significant main effect of trial type (compatible vs. incompatible), *F*(1, 28) = 24.31, *p* < 0.001, *η*_p_^2^ = 0.465, and a significant interaction between type of compatibility and trial type, *F*(1, 28) = 6.37, *p* < 0.05, *η*_p_^2^ = 0.185, but no significant main effect of type of compatibility (spatial vs. imitative), *F*(1, 28) = 3.06, *p* = 0.091, *η*_p_^2^ = 0.099.

A simple main-effects analysis of the interaction showed that the mean RT in the compatible trial was significantly faster than that in the incompatible trial in the spatial condition, *F*(1, 28) = 22.99, *p* < 0.001, *η_p_*^2^ = 0.451, but not in the imitative condition, *F*(1, 28) = 3.88, *p* = 0.087, *η_p_*^2^ = 0.122. Additionally, there was a significant simple main effect of type of compatibility (spatial vs. imitative) in the compatible trial, *F*(1, 28) = 7.91, *p* < 0.01, *η_p_*^2^ = 0.220, but no significant simple main effect of type of compatibility in the incompatible trial, *F*(1, 28) = 0.19, *p* = 0.683, *η_p_*^2^ = 0.007.

These results revealed that the mean RT in the compatible trial was faster than that in the incompatible trial in the spatial condition but not in the imitative condition. This finding suggests that the observation of light flashing in a sequential manner elicits a spatial compatibility effect but that the imitative compatibility effect is reduced in situations with a non-human stimulus. Furthermore, the imitative compatibility effect was larger with the human model (effect size in Experiment 1: *η_p_*^2^ = 0.266) than with the light (effect size: *η_p_*^2^ = 0.122), suggesting a social basis for imitation.

The mean error proportion for each type of compatibility and each trial type was extremely low (< 1%). The error data were subjected to a repeated-measures ANOVA with the same factors as the RT data. The results showed no significant main effects or interactions, *F*(1, 28) < 1.29, *p* > 0.71, *η_p_*^2^ < 0.044.

## 5. General discussion

Three experiments were used to examine the degree that automatic imitation occurs when methods adopt more complex movements than simple finger movements, such as finger lifting, in the delay paradigm. Across the first two experiments, participants responded more quickly in compatible movement trials than in incompatible trials, both in spatial and imitative conditions, regardless of whether the response depended on hand or foot movements. In Experiment 3, the imitative compatibility effect was weak for non-human actors, but the spatial compatibility effect persisted.

In short, a clear spatial compatibility effect (faster responses in compatible trials) was found in the RT data across the three experiments, regardless of whether the participants observed human actions or lights flashing. These findings suggest that the effect is driven by spatial processing that detects the similarity of spatial locations (e.g., keys or pedals) in the field of view and not by the similarity of body movements between the actor and the observer.

Furthermore, an imitative compatibility effect occurred when participants observed the movements of human models but not when they observed lights that flashed to represent the movements. Several previous studies using imitation–inhibition tasks have reported that non-human stimuli (e.g., a wooden hand, robotic hand, or moving dot) elicit a weaker imitative compatibility effect than do human stimuli (Brass et al., 2001; Press et al., 2006; Gowen et al., 2016). The current results are consistent with these findings. This suggests that the imitative compatibility effect is sensitive to similarities in appearance and biological movement. In contrast to the non-human stimuli used in previous studies, the non-human stimuli in this study had neither biological movements nor a human form. This could have resulted in participants being unable to feel connected to the stimulus, which could have contributed to the absence of an imitative compatibility effect.

The first two experiments show that imitative compatibility is not limited to hand-and-arm movements, but extends to foot-and-leg movements with human stimuli. These findings extend those of Pan and Hamilton (2015), who showed that imitative compatibility of hand-and-arm movements occurred with virtual characters. That is, the imitative compatibility exists between human stimuli and different effectors. Many previous studies of automatic imitation using simple finger or hand movements have used tasks in which participants performed the same or different movements while watching another person’s movements. By contrast, the current results show that automatic imitation occurs after watching these movements. Therefore, a novel and important implication of this study is that action observation automatically promotes the same response in an observer and memory works to maintain the automatic imitation effect.

The finding that the imitative compatibility effect was weak when participants observed flashing lights suggests that imitative compatibility in serial movements is not a byproduct of simple spatial processing. Sequential actions can elicit automatic imitation, in which participants imitate the observed sequential actions without awareness. These results have several implications for the psychological mechanisms underlying automatic imitation of sequential actions. First, the visual input of the movement sequences, which are task-irrelevant stimuli, automatically activates a visual representation of the movement. Second, the activated sensory representation leads to priming of the connected motor representation. Finally, activation of the corresponding motor representation leads to slower responses in incompatible trials than in compatible trials. Although these psychological mechanisms are speculative, they are consistent with the influential ASL model of imitation (Heyes, 2001, 2005, 2011), which proposes that the imitative compatibility effect results in bidirectional connections between the sensory (visual) and motor representations of the same action component. Both ideomotor theory and the ASL model emphasize the importance of associative learning in linking the visual representation of an action to its motor representation, and they assume that long-term stimulus–response associations mediate the automatic imitation of actions (Brass and Heyes, 2005; Heyes, 2011; Cracco et al., 2018). In particular, the ASL model assumes not only linkage between visual and motor representations of simple actions but also linkage between action sequences (Heyes, 2005), suggesting that the imitative compatibility effect in this study could result from an automatic activation of the observed action sequences in a similar way to simple actions. However, this study used a between-subjects design, and it is possible that at least some individual differences regarding imitative tendencies could have been introduced into the results. Therefore, it will be necessary in the future study to examine the results with a within-subjects design.

In contrast to RT data, this study found error rates (i.e., all <1%) that were much smaller than those in previous imitation–inhibition task studies. RT data are typically more sensitive to automatic imitation than are error data because the number of errors is typically low (i.e., < 5%) in the imitation–inhibition task (Cracco et al., 2018). However, there were no significant effects in the error data in this study. The experimental setup of this study could have encouraged participants to plan and control their movements, which could have led to fewer errors. Further research is needed to clarify the absence of compatibility effects in error data in observation-execution tasks.

Despite its implications, this study has several limitations. First, it is unknown whether the strong competition between self and other representations typically observed in traditional imitation–inhibition tasks might be fully at work in the delay paradigm employed in this study. Specifically, the low error rates suggest the possibility that the participants could suppress automatically activated motor representations of the model (i.e., others) in some extent when generating their own bodily movements. Alternatively, the current results could be explained by a priming effect stemming from participants’ attention to key or pedal positions and associated numbers while observing the stimulus movies, rather than imitative processes. In contrast to the simple finger or hand movements performed by isolated hands, the sequential movements utilized in this study were relatively high mental load when participants attempted to memorize them. To alleviate this mental load, it is conceivable that other processes such as verbalization could be involved. These considerations raise questions about the duration of the activation of motor representations induced by observing others’ actions and how long they might be retained as implicit memory. Recent research on implicit memory has reported fairly long retention periods (Mitchell et al., 2018); however, it remains a subject for future investigation whether motor memories formed through the observation of others’ movements persist over an extended period and influence one’s own motor output as automatic imitation or not.

Second, participants’ subvocalization of the order of pressing keys or stepping on foot pedals could have contributed to the imitative compatibility effect. If the participants used verbal encoding of the order in both the spatial and imitative conditions, one would expect no significant difference in the mean RT in the compatible trials between the spatial and imitative conditions. Although the results of Experiment 2 (foot-and-leg movement task) were consistent with this prediction, A significant simple main effect of the type of compatibility (spatial vs. imitative) in the compatible trial in both Experiment 1 (the hand-and-arm movement task) and Experiment 3 (the light-flashing task) was found. This suggests that observing a sequential action automatically influences subsequent performance. Perhaps there was no simple main effect in the foot-and-leg movement task because it would be relatively difficult for the participants to imagine the movements of their feet compared to those of their hands. Therefore, they could have tried to subvocalize the numbers of the presented sequences to reduce the cognitive load of memorizing the order. However, future work should consider ways to reduce the contamination of the verbal encoding of action sequences, perhaps by using symbolic cues instead of number cues.

Third, the sample size was smaller than that of other imitation–inhibition task studies, some of which were statistically high-power experiments with over 100 participants (Genschow et al., 2017; Westfal et al., 2021). Therefore, the lack of an imitative compatibility effect with light-flashing trials in Experiment 3 could have represented type II error due to the small sample size. Therefore, one should be careful when interpreting the results of Experiment 3. Executing a large-scale study was impossible because of the limitations of available resources. Future research should test the effects of this study using larger sample sizes to better estimate the true magnitude of the effects reported here.

In summary, the results suggest that automatic imitation is not limited to simple finger movements but also occurs when methods adopt more complex movements and more realistic motion profiles. These findings help account for when and how imitative behavior occurs, even when the imitator does not intend to copy the actions of other people. An ongoing question in psychology concerns the extent that behavior is controlled by intention and reason (Heyes, 2011; Genschow and Groß-Bölting, 2021). Therefore, work on automatic imitation of serial movements could play a crucial role in elucidating how individuals interpret other people’s actions. Moreover, this study illuminates the extent that people influence others’ behavior outside of intention or reason. These issues pertain to the overlap between experimental psychology, cognitive neuroscience, and experimental social psychology. Future studies should therefore encompass all three areas to reveal the psychological functions of the neural substrates involved in automatic imitation and to explain how imitation and being imitated promote prosocial behavior.

## Data availability statement

The original contributions presented in the study are included in the article/supplementary material, further inquiries can be directed to the corresponding author.

## Ethics statement

The studies involving humans were approved by the ethics board of Aichi University. The studies were conducted in accordance with the local legislation and institutional requirements. The participants provided their written informed consent to participate in this study. The individual(s) provided their written informed consent for the publication of any identifiable images or data presented in this article.

## Author contributions

HI: conceived and designed the experiments, performed the experiments, analyzed the data, and wrote the paper.
